# The expression of TLR2 and TLR4 in the kidneys and heart of mice infected with *Acanthamoeba* spp.

**DOI:** 10.1186/s13071-020-04351-4

**Published:** 2020-09-21

**Authors:** Karolina Kot, Danuta Kosik-Bogacka, Agnieszka Wojtkowiak-Giera, Agnieszka Kolasa-Wołosiuk, Natalia Łanocha-Arendarczyk

**Affiliations:** 1 Department of Biology and Medical Parasitology, Pomeranian Medical University in Szczecin, Powstancow Wielkopolskich 72, 70-111 Szczecin, Poland; 2 Independent of Pharmaceutical Botany, Department of Biology and Medical Parasitology, Pomeranian Medical University in Szczecin, Powstancow Wielkopolskich 72, 70-111 Szczecin, Poland; 3 Department of Biology and Medical Parasitology, Poznan University of Medical Sciences, Fredry 10, 61-701 Poznan, Poland; 4 Department of Histology and Embryology, Pomeranian Medical University in Szczecin, Powstancow Wielkopolskich 72, 70-111 Szczecin, Poland

**Keywords:** *Acanthamoeba* spp., Kidneys, Heart, Toll-like receptor 2 (TLR2), Toll-like receptor 4 (TLR4)

## Abstract

**Background:**

*Acanthamoeba* spp. are cosmopolitan protozoans that cause infections in the brain, as well as extracerebral infections in the cornea, lungs and skin. Little is known about the mechanisms of the immunological response to these parasites in organs which are not their main biotope. Therefore, the purpose of this study was to determine the expression of TLR2 and TLR4 in the kidneys and heart of *Acanthamoeba* spp.-infected mice, with respect to the host’s immunological status.

**Methods:**

The mice were grouped into four groups: immunocompetent control mice; immunosuppressed control mice; immunocompetent *Acanthamoeba* spp.-infected mice; and immunosuppressed *Acanthamoeba* spp. infected mice. In the study, we used the amoebae T16 genotype which was isolated from a patient. The TLRs expressions in the kidneys and heart of mice were assessed by quantitative real-time polymerase chain reaction. Moreover, we visualized TLR2 and TLR4 proteins in the organs by immunohistochemical staining.

**Results:**

In the kidneys, we observed a higher TLR2 expression in immunosuppressed mice at 24 days post-*Acanthamoeba* spp. infection (dpi) compared to the uninfected mice. There were no statistically significant differences in TLR4 expression in the kidneys between the immunocompetent and immunosuppressed mice, both of infected and uninfected mice. In the heart, we observed a difference in TLR2 expression in immunocompetent mice at 24 dpi compared to immunocompetent mice at 8 dpi. The immunocompetent *Acanthamoeba* spp.-infected mice had higher TLR4 expression at 8 dpi compared to the immunocompetent uninfected mice.

**Conclusions:**

Our results indicate that TLR2 is involved in response to *Acanthamoeba* spp. infection in the kidneys, whereas in the heart, both studied TLRs are involved.
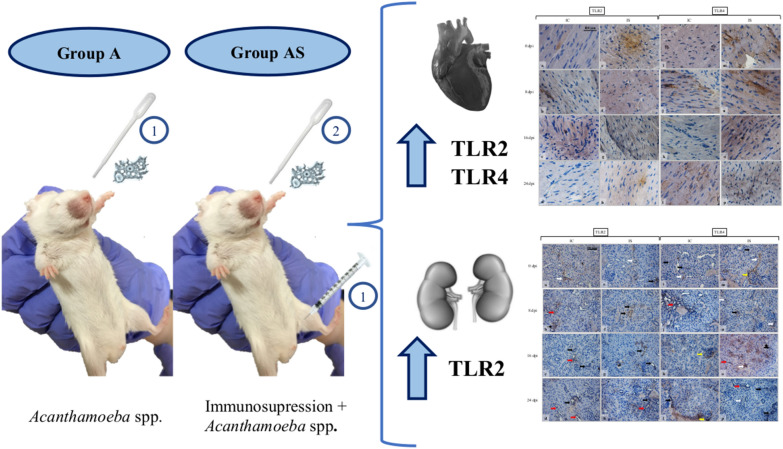

## Background

Acanthamoebiasis caused by protozoans of the genus *Acanthamoeba* is an infection that is more frequently found in patients with a low immune response. Development of this opportunistic infection is enhanced by chronic stress, coexisting diseases, and immunosuppressive drugs which are used to inhibit the rejection of transplanted organs [1]. It has been shown that corticosteroid therapy increases the number of parasites in the host [2]. Due to immunosuppression of the host organism, some parasitoses from asymptomatic or scarcely symptomatic infections leads to disseminated parasitic infections. These sudden multi-organ and multi-symptomatic changes may lead to the patient’s death [3].

*Acanthamoeba* spp. trophozoites enter into the tissue or organs and induce granulomatous amoebic encephalitis (GAE), *Acanthamoeba* keratitis (AK) or disseminated acanthamoebiasis [3, 4]. Disseminated acanthamoebiasis is predominantly confirmed *post-mortem* by histopathological examination of the organs and/or re-isolation of amoebae from tissue fragments because biochemical and hematological tests are difficult to interpret; blood parameters may be elevated, lower or not deviating from the norm [5, 6]. In our earlier study concerning hematological and biochemical profiles in the blood of experimentally *Acanthamoeba* spp.-infected mice, we found only higher level of lymphocytes, monocytes, thrombocytes and aspartate aminotransferase [7, 8], despite the re-isolation of these amoebae from the brain, eyeball, lungs, kidneys, and spleen of the mice [7–11].

Mechanisms of renal and heart infection in acanthamoebiasis are still unknown. The immune response in renal and cardiac muscle cells to circulating antigens can be augmented by Toll-like receptors (TLRs), which play a key role in the non-specific immune response, recognizing pathogen-associated molecular patterns (PAMPs) present in pathogenic microorganisms [12]. The PAMPs on the membrane of *Acanthamoeba* spp. comprise proteins (33%), phospholipids (25%), sterols (13%) and lipophosphoglycan (29%) [13]. Moreover, TLR-signaling pathways might also be activated by components discharged by tissue damage or inflammation, or damage-associated molecular patterns (DAMPs), and heat-shock proteins (HSP) -60 and -70, which are revealed in *Acanthamoeba* spp. [14]. The recognition of these patterns by TLRs initiates the migration and aggregation of immunes cells at the site of infection, which in turn leads to the development of inflammation [15]. TLRs activate signaling pathways that lead to activation of the transcription factors NF-κB and IRFs, which control the expression of genes encoding proinflammatory cytokines, such as TNF- α, IL-8 and IFN-γ [16, 17]. A lack of TLR2 and TLR4 on the immune cells leads to a delayed phagocytosis of pathogens, including bacteria such as *Escherichia coli*, *Salmonella typhimurium* and *Staphylococcus aureus* [18].

TLRs are involved in the response to several parasites, including *Acanthamoeba* spp. Alizadeh et al. [19] reported that *Acanthamoeba* spp. is recognized by TLR4, whereas other authors observed changes in TLR2 and TLR4 expression in the brain, lungs and eyes of *Acanthamoeba* spp.-infected mice [11, 20, 21]. However, no data exist on TLR activation in the organs and tissues which are not the main biotope of these protozoans. Therefore, the purpose of this study was to determine the TLR2 and TLR4 expression in the kidneys and heart of *Acanthamoeba* spp.-infected mice, with respect to the host’s immunological status.

## Methods

### Isolation of *Acanthamoeba* spp.

The *Acanthamoeba* spp. (AM22 strain) used in the study was isolated in 2009 from the bronchoaspirate fluid of a man with chronic leukaemia and atypical pneumonia. The patient had respiratory efficiency and acute septic shock. Moreover, interstitial changes with a visible pulmonary swelling were observed in the radiological examination. The AM22 strain was examined by molecular methods and the T16 genotype was detected [22]. Amoebae were kept on non-nutrient agar (NN agar) plates. Before the experiment, *Acanthamoeba* spp. were grown on NN agar plates with deactivated bacteria and incubated for 72 h at 37 °C [9].

### Animal model

The study was conducted on 96 male BALB/c mice about 6–10 weeks-old and an average weight of 23 g. Mice were obtained from a licensed breeder (the Center of Experimental Medicine, Medical University in Białystok, Poland). During the experiment, the animals were in the Animal Facility of the Pomeranian Medical University in Szczecin, where they had constant access to fresh, clean water and Labofeed feed (Morawski, Kcynia, Poland), following recommended standards. Animals were housed in polycarbonate cages, and they were kept in standard laboratory conditions in a cycle of 12 h of light/12 h of darkness, at a temperature of 22 ± 2 °C, humidity of 56% and 15–20 air changes per hour.

The mice were divided into four groups: (i) immunocompetent uninfected mice: immunocompetent control group (C, *n *= 18); (ii) immunocompetent *Acanthamoeba* spp.-infected mice (A, *n *= 30); (iii) immunosuppressed by methylprednisolone sodium succinate uninfected mice: immunosuppressed control group (CS, *n *= 18); and (iv) immunosuppressed by methylprednisolone sodium succinate *Acanthamoeba* spp.-infected mice (AS, *n *= 30).

Animals belonging to groups AS and CS were given 0.22 mg methylprednisolone sodium succinate (MPS; Solu-Medrol, Pfizer, Kent, UK) dissolved in 0.1 ml 0.9% saline. MPS was administered intraperitoneally daily for 4 days prior to *Acanthamoeba* spp. infection. The drug was given to reduce immunity of the mice [11, 23]. Animals belonging to groups A and AS were administered intranasally 3 μl of suspension consisting of 10,000–20,000 trophozoite form of *Acanthamoeba* spp. Mice from the control groups (C and CS) were given the same volume of saline. The animals were sacrificed by pentobarbital sodium (Euthasol vet, FATRO, Raamsdonksveer, The Netherlands), administered at 2 ml/kg mg/kg body weight intraperitoneally at 8, 16, and 24 days post-*Acanthamoeba* spp. infection (dpi). The kidneys and heart were collected from the mice.

### TLRs expression

TLR2 and TLR4 gene expression in the kidneys and heart of mice was assessed by quantitative real-time polymerase chain reaction using a Light Cycler real-time PCR detection system (Roche Diagnostic GmbH, Mannheim, Germany) as recently described [11, 20]. Briefly, the increase in reaction product was assessed by increasing the fluorescence signal of SYBR Green I dye binding to double-stranded DNA. Target cDNA was quantified using a relative estimation method using a calibrator, which was prepared as a cDNA mix from all samples, and subsequent dilutions were used to create a standard curve according to the manufacturer’s instructions (Roche Diagnostic GmbH). The housekeeping gene porphobilinogen deaminase was amplified as the reference gene for mRNA quantification. The amount of TLR2 and TLR4 transcripts in each sample was determined by the geometric mean of the transcript level of the housekeeping gene, and the mRNA levels of TLR2 and TLR4 are expressed as the multiplicity of cDNA concentrations in the calibrator.

### Immunohistochemical staining

The paraffin-embedded sections (3–5 μm) of kidneys and heart of control mice and hosts infected with *Acanthamoeba* spp. were stained to visualize TLR2 and TLR4 proteins. Immunohistochemistry was performed using specific primary rabbit polyclonal antibodies against TLR2 and TLR4 (Cat# sc-10739 and sc-30002, respectively; Santa Cruz Biotechnology, Inc., Oregon, USA) at a 1:500 dilution. The procedure of immunohistochemical staining is described by Kot et al. [11]. Briefly, the sections were microwaved to recover antigenicity and then incubated with primary antibodies overnight at 4 °C. Subsequently, the sections were stained with an avidin-biotin-peroxidase system with diaminobenzidine as the chromogen (Cat# K0679; DakoCytomation Inc., Carpinteria, CA, USA). The sections were washed in distilled H_2_O and counterstained with hematoxylin. In negative controls, samples were not incubated with primary antibodies. Positive samples were determined microscopically by identifying brown pigmentation. Samples were evaluated using a light microscope (DM5000B; Leica, Wetzlar, Germany).

### Statistical analysis

Statistical analysis was made using Microsoft Excel 2016 and StatSoft Statistica v10.0. Comparisons between intergroups were made using the Mann-Whitney U-test. The level of significance was *P *< 0.05.

## Results

### TLR2 and TLR4 expression in the kidneys

**Fig. 1:**
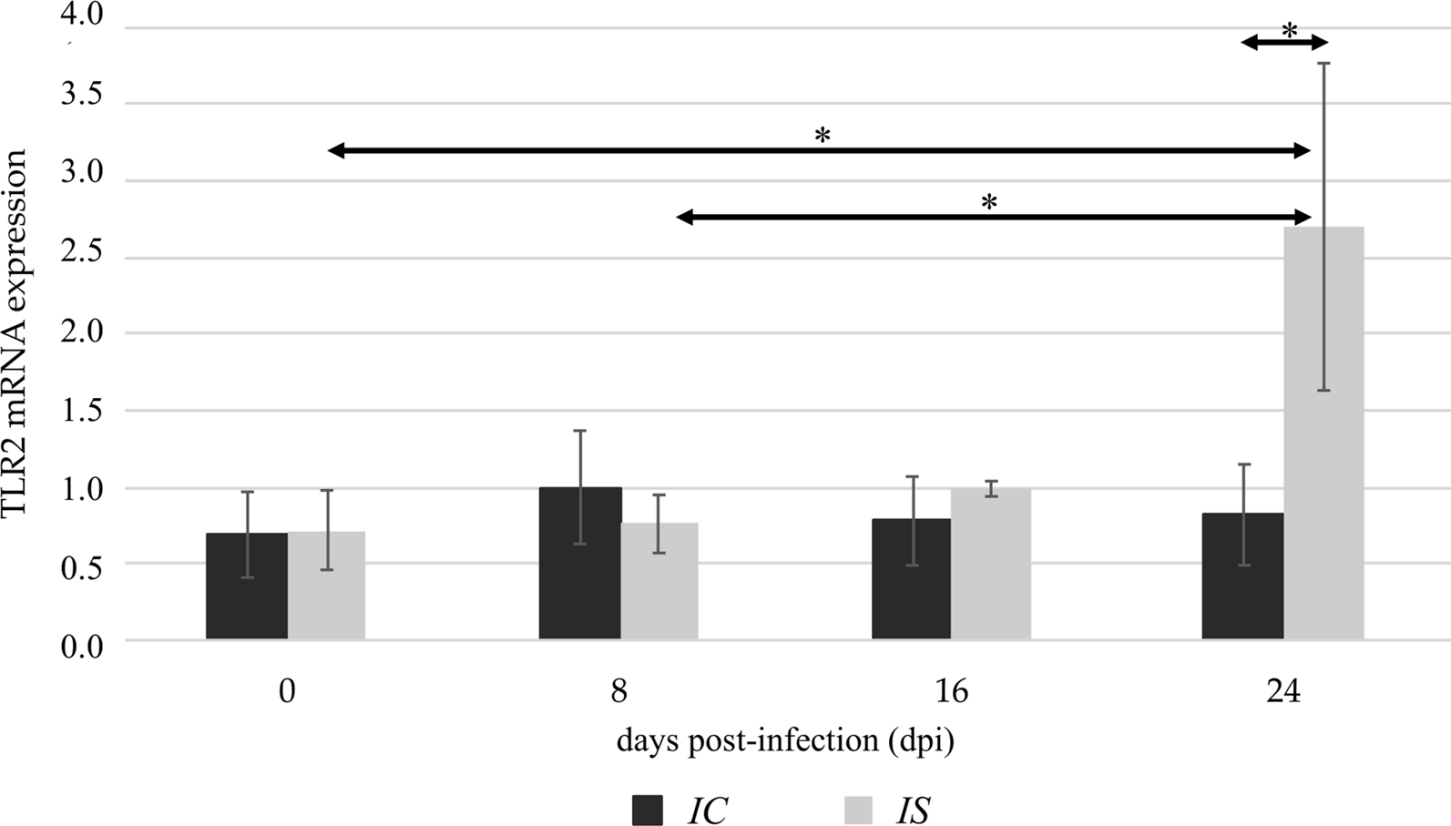
The TLR2 mRNA expression in the kidneys of uninfected (0 dpi) and infected mice at 8, 16, and 24 dpi, in accordance with the immunological status of hosts (IC, immunocompetent mice; IS, immunosuppressed mice). The results are the means and standard deviations (SD) of six independent experiments; *P < 0.05 using a Mann-Whitney U-test

**Fig. 2:**
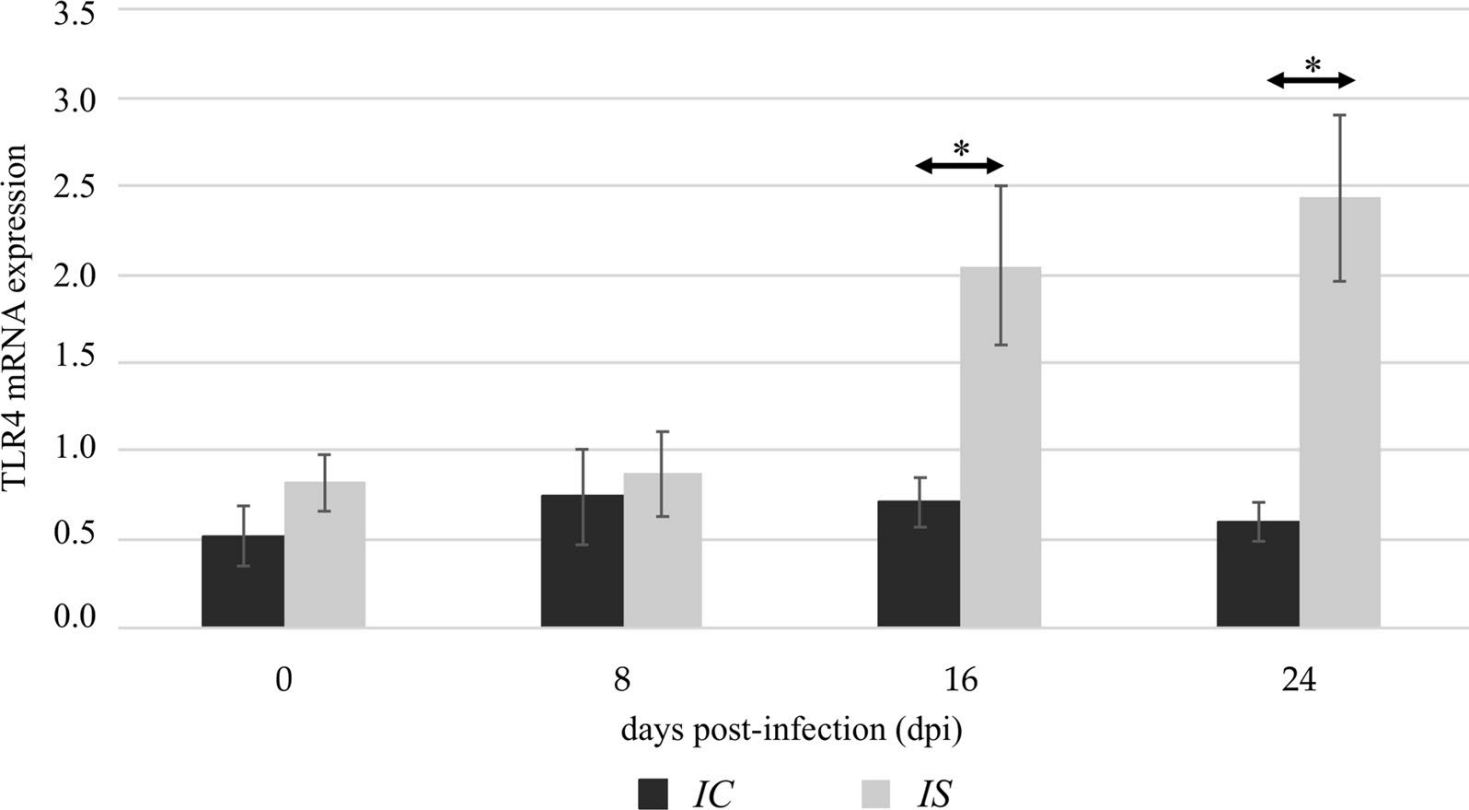
The TLR4 mRNA expression in the kidneys of uninfected (0 dpi) and infected mice at 8, 16, and 24 dpi, in accordance with the immunological status of hosts (IC, immunocompetent mice; IS, immunosuppressed mice). The results are the means and standard deviations (SD) of six independent experiments; *P < 0.05 using a Mann-Whitney U-test

The TLR2 expression in the kidneys of immunocompetent mice was similar to uninfected and *Acanthamoeba* spp.-infected mice. In immunosuppressed mice, we found a significantly higher level of TLR2 mRNA expression at 24 days post-*Acanthamoeba* spp. infection (dpi) compared to uninfected animals (0 dpi; *U* = 24, *P* < 0.05), and the levels obtained from mice at 8 dpi (*U* = 16, *P* < 0.05; Fig. 1). Additionally, there was a statistically significant difference in the TLR2 expression between the immunocompetent and immunosuppressed *Acanthamoeba* spp.-infected mice at 24 dpi (*U* = 20, *P* < 0.05; Fig. 1). The TLR4 expressions in the immunocompetent uninfected mice and mice at 8 dpi, 16 dpi and 24 dpi were similar. The TLR4 mRNA expression in the immunosuppressed *Acanthamoeba* spp.-infected mice was higher at 16 dpi and 24 dpi compared to the immunosuppressed uninfected mice, but the differences were not statistically significant. Taking into account the immunological status of the hosts, we found statistically significant differences in the TLR4 expression between the immunocompetent and immunosuppressed mice at 16 dpi and at 24 dpi (*U* = 12 and *U* = 15, *P* < 0.05, respectively; Fig. 2).

**Fig. 3:**
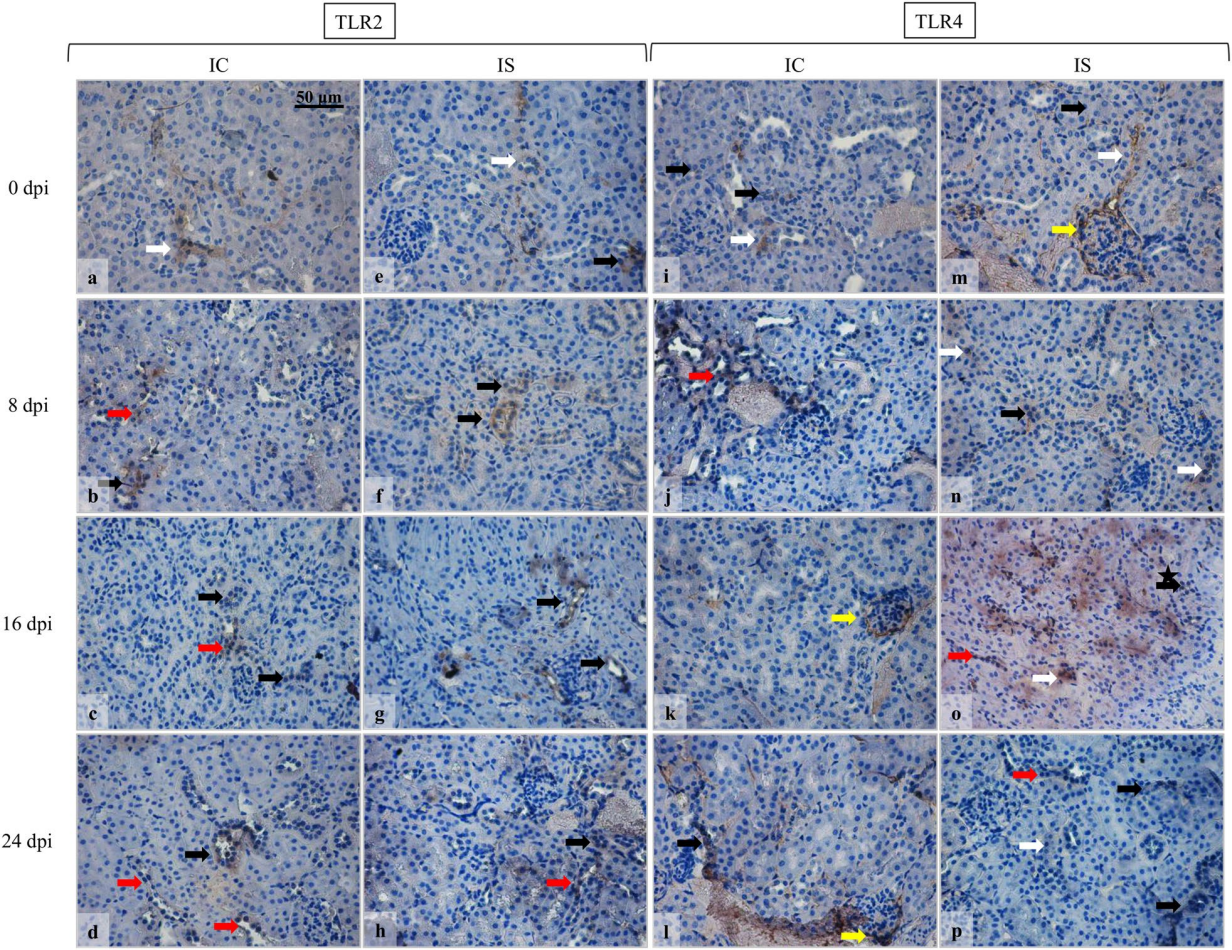
Immunohistochemical staining with primary anti-TLR2 (**a**–**h**) and anti-TLR4 antibodies (**i**–**p**) in the kidneys of immunocompetent and immunosuppressed mice from control groups (0 dpi) and at 8, 16, and 24 dpi. Magnification 40×. In the immunocompetent and immunosuppressed uninfected mice, TLR2 expression was found in the proximal tubules (white arrows) (**a**, **e**), while after *Acanthamoeba* spp. infection brown pigmentation was observed in the distal tubules and collecting ducts (black and red arrows, respectively) (**a**–**h**). The immunoexpression of TLR4 was observed in the proximal and distal tubules (white and black arrows, respectively), collecting ducts (red arrows) and in the renal corpuscles (yellow arrows) (**i**–**p**). The immunosuppressed mice after 16 days post-*Acanthamoeba* spp. infection had immunopositive nuclei in the epithelial cells of the distal tubule (black asterisk) and the highest intensity of TLR4 immunoreaction the intensity of TLR4 immunoreaction (**o**). *dpi* days post-*Acanthamoeba* spp. infection, *IC* immunocompetent mice, *IS* immunosuppressed mice

The results of the immunohistochemical reaction showed changes in the intensity of TLR2 and TLR4 immunoexpression in the kidneys of *Acanthamoeba* spp.-infected mice in comparison to the control groups (Fig. 3). In the immunocompetent and immunosuppressed uninfected mice, TLR2 expression was found in the proximal tubules (Fig. 3, white arrows), while after *Acanthamoeba* spp. infection brown pigmentation was observed in the distal tubules and collecting ducts (Fig. 3, black and red arrows, respectively). The TLR2 expression in the kidneys of the immunocompetent *Acanthamoeba* spp.-infected mice remained at a similar level during the infection. In the immunosuppressed mice, the TLR2 expression intensity and the number of immunopositive cells were similar at 8 dpi and 16 dpi (Fig. 3). During the decline of infection, in the 24 dpi group TLR2 expression increased. The immunoexpression of TLR4 was observed in the proximal and distal tubules (Fig. 3, white and black arrows, respectively), collecting ducts (Fig. 3, red arrows) and in the renal corpuscles (Fig. 3, yellow arrows). The TLR4 expression in the immunocompetent *Acanthamoeba* spp.-infected mice was similar to TLR2 expression and remained at a similar level during the infection. In the immunosuppressed *Acanthamoeba* spp.-infected mice, the number of TLR4 immunopositive cells and the intensity of TLR4 immunoreaction increased at 16 dpi and then decreased at 24 dpi (Fig. 3). The highest intensity of TLR4 immunoreaction was observed in the immunosuppressed mice at 16 days post-*Acanthamoeba* spp. infection.

### TLR2 and TLR4 expressions in the heart

**Fig. 4:**
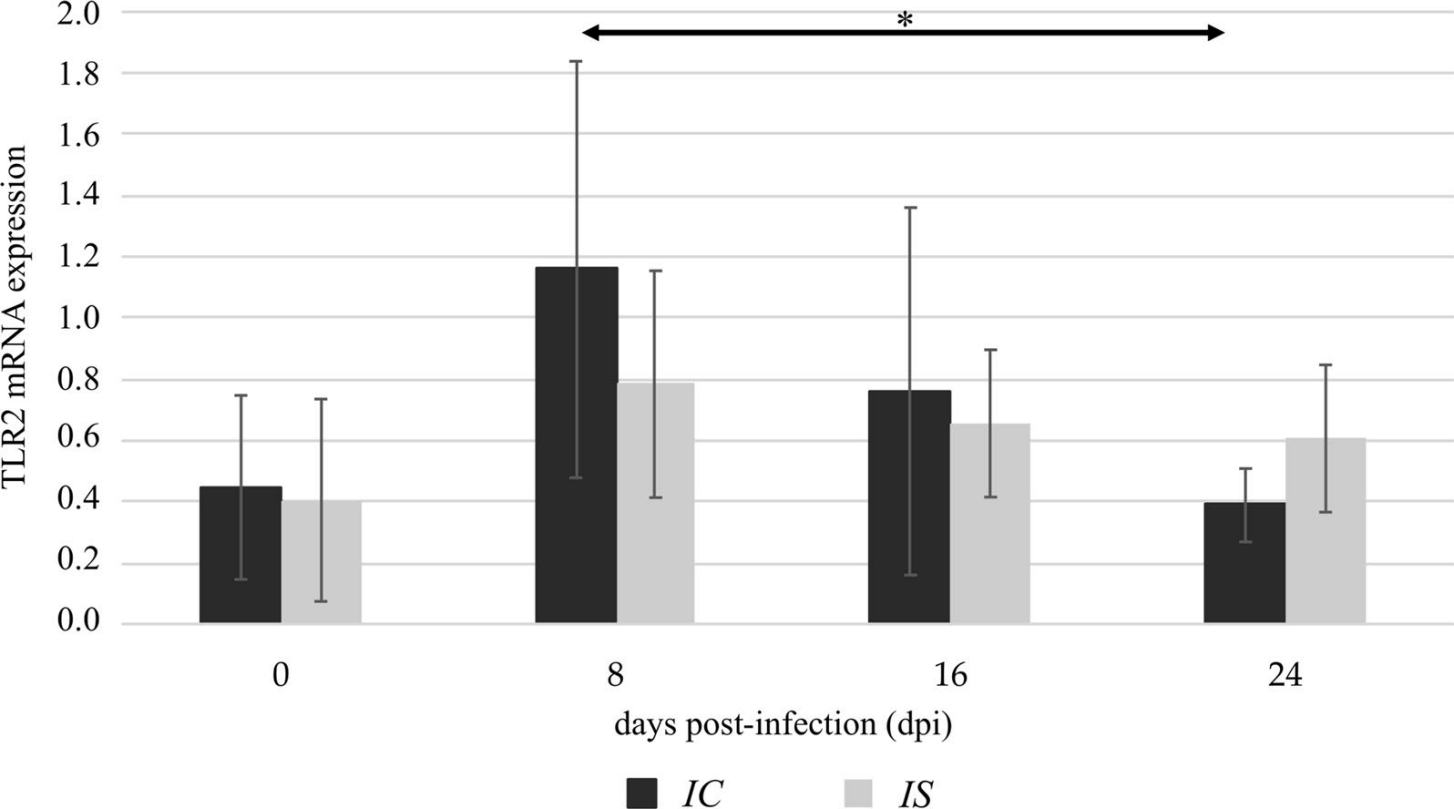
The TLR2 mRNA expression in the heart of uninfected (0 dpi) and infected mice at 8, 16, and 24 dpi, in accordance with the immunological status of hosts (IC, immunocompetent mice; IS, immunosuppressed mice). The results are the means and standard deviations (SD) of six independent experiments; *P < 0.05 using a Mann-Whitney U-test

**Fig. 5:**
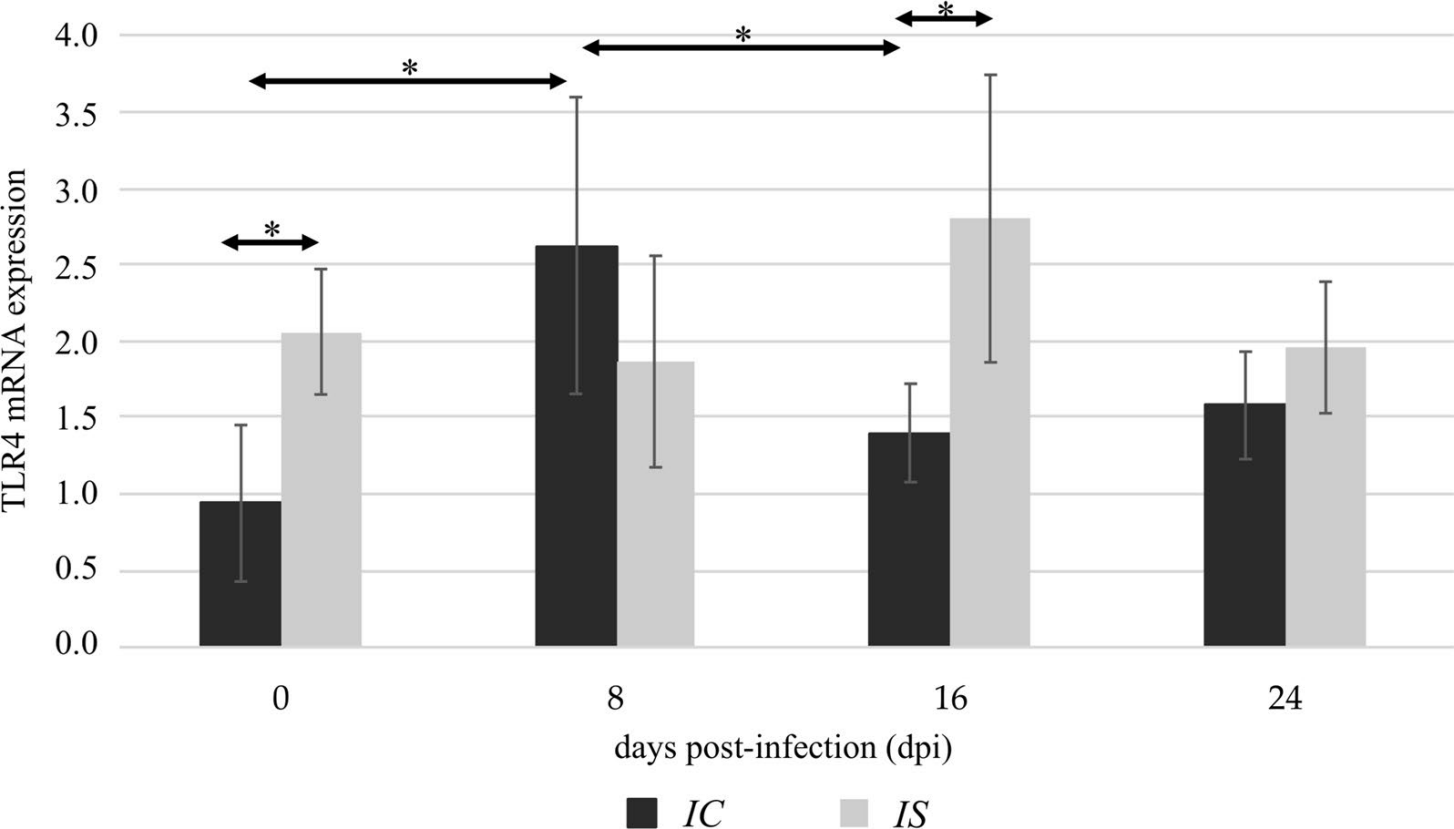
The TLR4 mRNA expression in the heart of uninfected (0 dpi) and infected mice at 8, 16, and 24 dpi, in accordance with the immunological status of hosts (IC, immunocompetent mice; IS, immunosuppressed mice). The results are the means and standard deviations (SD) of six independent experiments; *P < 0.05 using a Mann-Whitney U-test

In the heart of the immunocompetent *Acanthamoeba* spp.-infected mice, the highest TLR2 expression was observed at 8 dpi. In the following days, TLR2 expression decreased. We found a statistically significant difference in the TLR2 expression level in the immunocompetent mice between 8 dpi and 24 dpi (*U* = 31.5, *P* < 0.05; Fig. 4). In immunosuppressed mice, no statistically significant differences were found in the TLR2 expression levels between infected and uninfected groups. The TLR4 expression was increased in immunocompetent mice at 8 dpi compared to uninfected animals and decreased in immunocompetent mice at 16 dpi compared to mice at 8 dpi (*U* = 23.5 and *U* = 22.5, *P* < 0.05, respectively; Fig. 5). In the immunosuppressed mice, there were no statistically significant differences in the TLR4 expression levels between *Acanthamoeba* spp.-infected and uninfected mice. Comparing the TLR4 immunoexpression between immunocompetent and immunocompromised mice, we found the differences in uninfected animals and in the mice at 16 days post-*Acanthamoeba* spp. infection (*U* = 23 and *U* = 23, *P* < 0.05, respectively; Fig. 5).

**Fig. 6:**
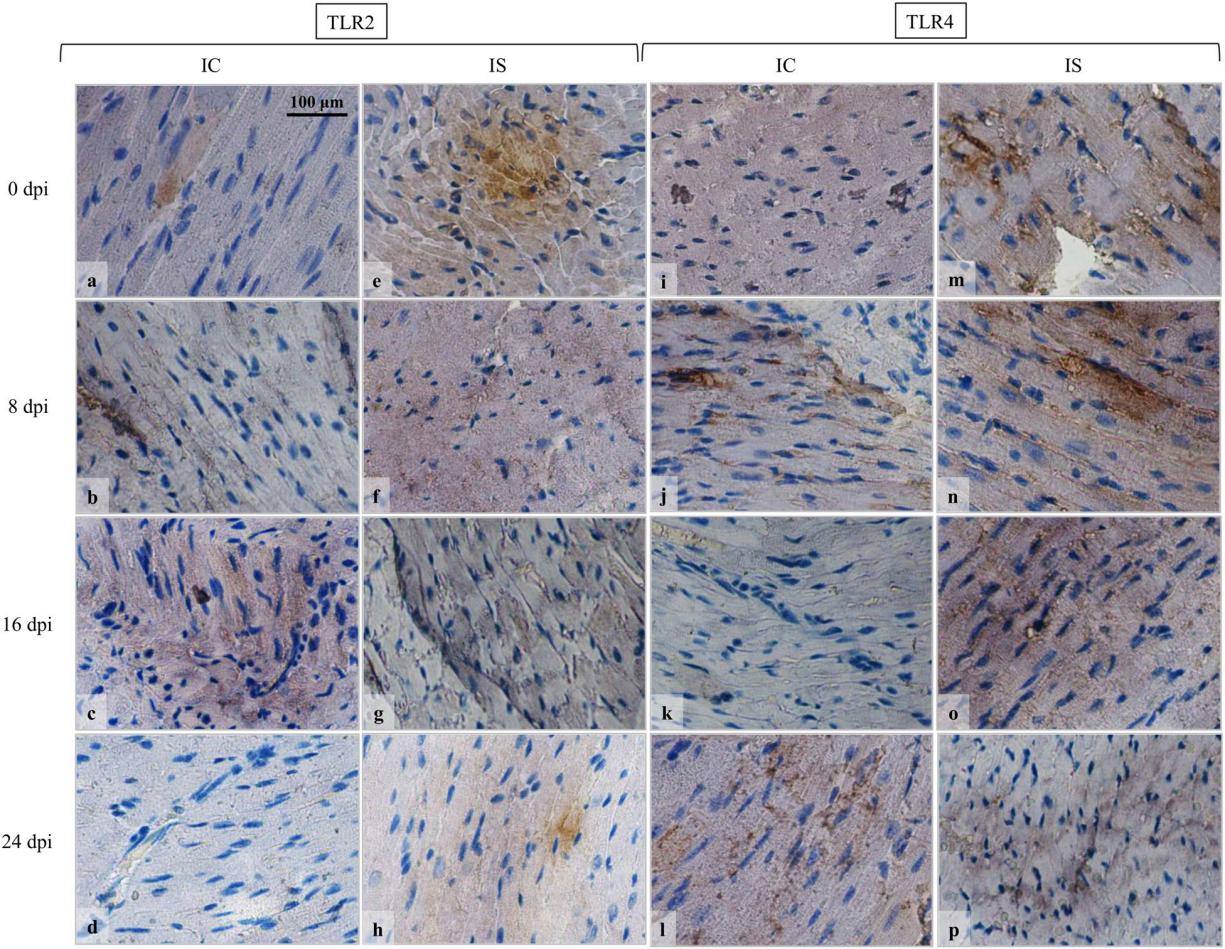
Immunohistochemical staining with primary anti-TLR2 (**a**–**h**) and anti-TLR4 antibodies (**i**–**p**) in the heart of immunocompetent and immunosuppressed mice from control group (0 dpi) and at 8, 16 and 24 dpi. Magnification 100×. In the immunocompetent and immunosuppressed control mice, only some cardiomyocytes showed TLR2 expression (**a**, **e**). At the beginning of infection in immunocompetent mice, TLR2 expression was observed in most cardiomyocytes (**b**, **c**). The lowest immunohistochemical reaction in the heart of immunocompetent mice was found at 24 dpi (**d**). In the immunosuppressed *Acanthamoeba* spp.-infected mice, the highest intensity of TLR2 expression was noted at 8 and 16 dpi (**f**, **g**). In the 24 dpi group of immunosuppressed mice, TLR2 was expressed only in some cardiomyocytes (**h**). In the heart of immunocompetent *Acanthamoeba* spp.-infected mice, increased TLR4 expression at 8 dpi was observed (**j**), while at 16 dpi, decreased number of TLR4-positive cells was found (**k**). In the immunosuppressed *Acanthamoeba* spp.-infected mice, the highest TLR4 expression was observed in the hearts of mice at 8 and 16 dpi (**n**, **o**), while at 24 dpi there was a decrease in TLR4 expression (**p**). *dpi* days post-*Acanthamoeba* spp. infection, *IC* immunocompetent mice, *IS* immunosuppressed mice

Immunohistochemistry revealed changes in the TLR2 and TLR4 immunoexpression in the heart of immunocompetent *Acanthamoeba* spp.-infected mice compared to uninfected mice (Fig. 6). In the immunocompetent and immunosuppressed control mice, only some cardiomyocytes showed TLR2 expression (Fig. 6a and 6e, respectively). At the beginning of infection in immunocompetent mice, TLR2 expression was observed in most cardiomyocytes (Fig. 6b, c). The lowest immunohistochemical reaction in the cardiac muscles of immunocompetent mice was found at 24 dpi (Fig. 6d). In the immunosuppressed *Acanthamoeba* spp.-infected mice, the highest intensity of TLR2 expression was noted at 8 and 16 dpi as brown pigmentation in the most cardiomyocytes (Fig. 6f and 6g, respectively). In the 24 dpi group of immunosuppressed mice, TLR2 was expressed only in some cardiomyocytes (Fig. 6h). We did not find TLR2 immunopositive cardiomyocyte nuclei in the hearts of immunocompetent and immunosuppressed *Acanthamoeba* spp.-infected mice (Fig. 6). In the heart of immunocompetent *Acanthamoeba* spp.-infected mice, we observed increased TLR4 expression at 8 dpi, while at 16 dpi, we observed decreased number of TLR4-positive cells (Fig. 6). In the immunosuppressed *Acanthamoeba* spp.-infected mice, the highest TLR4 expression was observed in the hearts of mice at 8 and 16 dpi, while at 24 dpi there was a decrease in TLR4 expression (Fig. 6p). The number of immunopositive cells and intensity of immunohistochemical reaction indicate a higher TLR4 expression in immunosuppressed uninfected mice than in immunocompetent uninfected mice (Fig. 6i, m).

## Discussion

The immune response against parasitic infection is complex and involves many effectors and regulators components. *Acanthamoeba* spp. activate the classical TLR signaling pathway inducing NF-kB activation and increased secretion of inflammatory cytokines [24]. Induction of an inflammatory response by amoebae has been proposed as an important factor to determine the course of the parasite infection [8]. Nevertheless, little is known about the innate immune response induced by *Acanthamoeba* spp. in the kidneys and heart. To our knowledge, this is the first report showing TLRs expression in the kidneys and heart of hosts with disseminated acanthamoebiasis. Hence, further knowledge about the molecular and immunological mechanisms induced by amoebae are important aspects to understand the course of infections and tissue invasion.

The occurrence of *Acanthamoeba* spp. infection has been described in patients following kidney transplantation [25–28], although in just one instance the amoebae were re-isolated from the kidney of a patient with probable *Acanthamoeba* meningoencephalitis [29]. In our experimental model, despite the re-isolation of *Acanthamoeba* spp. from the mouse kidneys, the kidney profile performed in mouse serum indicated normal renal function [7]. In the study concerning histopathological changes in the kidneys of mice infected with *Acanthamoeba* spp. strain AM22, we observed elevated proliferation of cellular nuclei in the proximal/distal tubule epithelium and areas with less acidic cytoplasm (unpublished data). In the kidneys of mice infected with an environmental isolate of *Acanthamoeba* spp., Górnik & Kuźna-Grygiel [30] observed haemorrhages, inflammatory foci, and even necrotic changes in the renal tubules and Bowman’s capsules. Therefore, based on previous studies, the affinity of *Acanthamoeba* spp. to the kidneys can neither be confirmed nor excluded.

The factors involved in kidney damage and abnormal kidney function in parasitic diseases are still unknown. Studies on visceral leishmaniasis associated with renal abnormalities, suggest that *Leishmania* spp. antigens induce kidney inflammation by activating TLR2 and TLR4 receptors. The results indicate that kidney inflammatory processes and apoptosis involving TGF-β have a significant role in the pathomechanism of kidney damage in *Leishmania donovani* infection [31]. However, based on studies on the role of Toll-like receptors in nephropathy induced by a *Toxoplasma gondii* infection, it was found that mainly TLR2 plays a role in kidney protection against *T. gondii* infection. Histopathological studies showed larger kidney damage in TLR2 deficient mice compared to TLR4 deficient mice [32]. In the present study, we found upregulation of both TLRs in immunocompromised *Acanthamoeba* spp.-infected mice, but statistically significant differences were only found in TLR2 expression on the decline of infection. Higher TLRs expression at 16 and 24 dpi may be a result of used corticosteroid or the number of parasites that entered the kidneys. Mun et al. [33] observed that the effect of TLR2 on survival of *T. gondii*-infected mice depended on the infection dose. In our study, the mice were infected with 10–20 thousand amoebae, but *Acanthamoeba* spp. were re-isolated mostly from the kidneys of immunosuppressed mice at 16 dpi and 24 dpi.

Leemans et al. [34] have shown that TLR2 plays a critical role in the initiation of acute renal inflammation and early tubular injury. Histological and morphological examination of *Acanthamoeba* spp.-infected kidneys revealed only elevated proliferation of nuclei in the renal tubules and a lighter color of the kidney parenchyma (unpublished data). It is possible that histopathological changes in the form of inflammatory infiltrates could have been visible in the kidneys of mice in a longer-lasting acanthamoebiasis; statistically significant changes in TLR2 expression were observed only at 24 dpi, the last day of the experiment. In future studies, the experiment should be extended to 30–40 days post-*Acanthamoeba* spp. infection and include determination of TGF-β expression which induces transformation of kidney tubule cells to proliferating fibroblasts, causing fibrous changes in kidney parenchyma [35]. Such changes may be visible in histological preparations in the form of a lighter color of the kidney parenchyma.

The current literature presents three cases of cardiovascular patients with acanthamoebiasis [6, 36, 37], although no parasite forms have been found in cardiac muscle cells [37]. Also, in this study no trophozoites or cysts were found in myocardial histological preparations from *Acanthamoeba* spp.-infected mice, despite the fact that developmental forms of *Acanthamoeba* spp. were isolated from these samples (unpublished data). Therefore, it is not clear whether the amoebae were re-isolated from cardiac fragments or from residual blood in the heart, as the amoebae spread throughout the host organism *via* the bloodstream.

The expression of Toll-like receptors has been reported in epithelium, endothelium and other cardiovascular cells [38, 39]. The results of studies conducted so far suggest that short-term activation of TLR receptors has a protective effect on the cardiovascular system, while long-term or excessive activation of these receptors induces chronic inflammation [38–40]. Cardiomyocytes in response to inflammatory stimuli are capable of secreting pro- and anti-inflammatory cytokines capable of initiating and regulating the inflammatory response, as well as chemokines, which recruit and activate appropriate inflammatory cells [41]. The data on TLR expression in the cardiac muscle cells of infected hosts are scarce. Ponce et al. [42] observed increased *tlr2* gene expression in the heart of BALB/c neonatal mice infected with *Trypanosoma cruzi*. The authors suggested that these parasites may activate the host’s innate immune response *via* different Toll-like receptors to protect cardiomyocytes from parasites. In contrast, Pereira et al. [43] suggest that a high TLR2 expression in patients with chronic Chagas cardiomyopathy may induce an increase in IL-1β, IL-12 and TNF-α, thereby elevating cardiac inflammation and contributing to heart dysfunction. Oliveira et al. [44] observed that a deficiency of TLR4 leads mice to being more susceptible to *T. cruzi* infection, as evidenced by a higher parasitemia and earlier mortality. However, it is important to point out that *T. cruzi* has an affinity for the heart, which is not the main biotope of *Acanthamoeba* spp. In the present study, a significant upregulation of TLR2 expression was seen in the heart of immunocompetent mice at the beginning of infection. Additionally, we observed a significant increase in TLR4 expression in the heart of immunocompetent mice at the beginning of infection, followed by a statistically significant reduction at 16 dpi. This shows that TLR2 and TLR4 induced an immune response at 8 days post-*Acanthamoeba* spp. infection and thus protected cardiomyocytes from parasites, as confirmed by histological studies which did not show morphological changes (unpublished data).

Lack of statistically significant changes in TLRs expression in the heart of immunosuppressed mice may be involved with used corticosteroid. Mogensen et al. [45] concluded that dexamethasone, an immunosuppressive drug, inhibits TLR-receptor signaling in *Neisseria meningitidis* and *Streptococcus pneumoniae* invasion. Ma et al. [46] examining cardiomyopathy in mice, found that blocking TLR2 activity blunted cardiac dysfunction and inhibited cardiac fibrosis, whereas blocking TLR4 exacerbated cardiac dysfunction and fibrosis. Histological studies of *Acanthamoeba* spp.-infected mice treated with an immunosuppressive drug showed morphological changes in the form of hemorrhages and vacuolized cardiomyocytes with less acidic cytoplasm at 8 dpi and 16 dpi. At 24 dpi, we observed no morphological changes in the cardiac muscle (unpublished data), which might have been caused by slightly increased TLR4 expression in mice treated with the immunosuppressive drug at 16 dpi.

## Conclusions

The immunological mechanisms preventing renal and cardiomyocyte pathomechanisms in *Acanthamoeba* spp. infection remain unknown. The present study showed upregulation in TLRs in the kidneys and heart of hosts with disseminated acanthamoebiasis. Our results indicate that TLR2 is involved in response to *Acanthamoeba* spp. infection in the kidneys, whereas in the heart, both studied TLRs are involved in response to *Acanthamoeba* spp. infection. In future studies, it will be important to analyze the cytokine profile in the heart and kidneys of hosts with disseminated acanthamoebiasis to better understand the course of infection and tissue invasion.

## Data Availability

The datasets used and analysed during the present study are available from the corresponding author upon reasonable request.

## Abbreviations

A: immunocompetent *Acanthamoeba* spp.-infected mice
AK: *Acanthamoeba* keratitis
AM 22: amoebic strain no. 22
AM: arithmetic mean
AS: immunosuppressed by methylprednisolone sodium succinate *Acanthamoeba* spp.-infected mice
C: immunocompetent uninfected mice, immunocompetent control group
CS: immunosuppressed by methylprednisolone sodium succinate uninfected mice, immunosuppressed control group
DAMPs: damage-associated molecular patterns
dpi: days post-infection
GAE: granulomatous amebic encephalitis
HSP60: heat shock protein 60
HSP70: heat shock protein 70
IC: immunocompetent mice
IS: immunosuppressed mice
IL-1β: interleukin 1β
IL-8: interleukin 8
IL-12: interleukin 12
IFN-γ: interferon gamma
IRF: interferon regulatory factor
MPS: methylprednisolone sodium succinate
NF-κB: nuclear factor kappa-light-chain-enhancer of activated B cells
NN: non-nutrient agar
*P*: level of significance
PAMPs: pathogen-associated molecular patterns
PBS: phosphate-buffered saline
SD: standard deviation
TLR2: toll-like receptor 2
TLR4: toll-like receptor 4
TGF- β: transforming growth factor β
TNF-α: tumor necrosis factor alpha
